# Association Between Birth Weight and Severity of Neonatal Jaundice: A Case-Control Study

**DOI:** 10.7759/cureus.93966

**Published:** 2025-10-06

**Authors:** Dunya Al-Rabeeah, Zainab Al-Majmaie, Ibrahim Saleem, Harith Al-Gburi

**Affiliations:** 1 Pediatric Surgery, Ibn Al-Balady Hospital, Baghdad, IRQ; 2 Diagnostic Radiology, Al Iraqia University College of Medicine, Baghdad, IRQ; 3 Internal Medicine, Salford Royal Hospital, Manchester, GBR; 4 General Surgery, Al Numan General Hospital, Baghdad, IRQ

**Keywords:** and hyperbilirubinemia, jaundice neonatal, low birth weight babies, pediatrics & neonatology, risk factors

## Abstract

Background

Neonatal jaundice (NNJ) is a common condition among newborns worldwide, with some cases progressing to severe hyperbilirubinemia and serious neurological complications. Identifying high-risk infants is critical for early intervention. While low birth weight (LBW) is widely recognized as a potential risk factor due to immature liver function and feeding issues, the association between macrosomia and NNJ remains unclear. Given regional variations in risk factors, this study aimed to assess the relationship between birth weight categories and the severity of NNJ in neonates admitted to Ibn Al-Balady Hospital in Baghdad, Iraq. The hypothesis was that LBW infants are at a higher risk of developing severe jaundice compared to those with normal birth weight.

Objective

To investigate the association between birth weight categories and the severity of neonatal jaundice among infants admitted to Ibn Al-Balady Hospital of Children and Maternity in Baghdad, Iraq.

Methods

A retrospective case-control study was conducted at Ibn Al-Balady Hospital in Baghdad from May 2 to June 22, 2025. Cases included neonates (≤28 days) diagnosed with severe neonatal jaundice requiring phototherapy or exchange transfusion, while controls were neonates without severe jaundice, matched at a 2:1 ratio. Data were extracted from medical records using a standardized form and included birth weight, gestational age, mode of delivery, maternal factors, and bilirubin levels. Birth weight was categorized as LBW (<2500 g), NBW (2500-3999 g), and macrosomia (≥4000 g). Statistical analysis involved chi-square tests, t-tests, and multivariate logistic regression to assess associations between birth weight and severe NNJ, adjusting for confounders like gestational age. Ethical approval was obtained, and patient confidentiality was maintained.

Results

A total of 152 cases with severe NNJ and 305 controls were included. The proportion of low birth weight infants was significantly higher in the severe jaundice group compared to controls (25.7% vs. 9.2%, p<0.001). After adjusting for gestational age, low birth weight was significantly associated with increased odds of severe NNJ [adjusted odds ratio (AOR)=2.78, 95% confidence interval (CI): 1.59-4.86, p<0.001]. No significant association was found between macrosomia and severe NNJ (Adjusted OR=1.15, 95% CI: 0.48-2.77, p=0.75).

Conclusion

Preterm birth and low birth weight are major risk factors for severe neonatal jaundice. Early identification and close monitoring of high-risk infants are crucial to prevent complications. Establishing national guidelines in Iraq would help standardize care and improve outcomes for affected newborns.

## Introduction

Neonatal jaundice (NNJ), characterized by yellowish discoloration of the skin and sclera due to elevated serum bilirubin levels, is one of the most common conditions requiring medical attention in newborns worldwide [1]. While often benign and resolving spontaneously, physiological jaundice in a significant proportion of infants can develop into severe hyperbilirubinemia, potentially leading to acute bilirubin encephalopathy (ABE) or chronic neurological impairment known as kernicterus [2-3]. The incidence and severity of NNJ vary globally, influenced by genetic factors, ethnic backgrounds, socioeconomic conditions, and healthcare practices [4].

Identifying infants at high risk for developing severe hyperbilirubinemia is paramount for implementing effective screening, surveillance, and timely intervention strategies like phototherapy or exchange transfusion [5-6]. Several risk factors have been established, including prematurity, blood group system (ABO)/Rh incompatibility, glucose-6-phosphate dehydrogenase (G6PD) deficiency, exclusive breastfeeding with excessive weight loss, sepsis, and a history of sibling jaundice [7-8].

Birth weight is another frequently considered factor. Low birth weight (LBW) was defined as a birth weight of less than 2500 grams (<2500 g). Infants, often associated with prematurity, may have immature hepatic enzymatic pathways for bilirubin conjugation and excretion, increased bilirubin production, and feeding difficulties contributing to increased enterohepatic circulation [9-10]. Conversely, the relationship between macrosomia (≥4000 g) and neonatal jaundice is less consistently reported in the literature. However, some studies suggest a potential association, possibly mediated by birth trauma, cephalohematoma, or delayed bilirubin clearance, cephalohematoma, or maternal diabetes [11-12].

While the general association between birth weight and jaundice is acknowledged, context-specific data are essential [13]. Healthcare systems, population genetics, and prevalent risk factors can vary significantly between regions. In Iraq, especially in a busy pediatric setting like Ibn Al-Balady Hospital in Baghdad, understanding the local epidemiology and risk factors for severe NNJ is essential for improving patient care pathways. Therefore, this study aimed to investigate the specific association between different birth weight categories and the severity of neonatal jaundice in infants admitted to this tertiary pediatric hospital. We hypothesized that neonates with lower birth weights would have significantly higher odds of developing severe jaundice compared to those with normal birth weights.

## Materials and methods

Study design and setting

This was a retrospective case-control study conducted at the Neonatal Care Unit (NCU) and pediatric wards of Ibn Al-Balady Hospital of Children and Maternity, Baghdad, Iraq. This is a major tertiary referral center serving a large population in Baghdad and the surrounding provinces. Data were collected from the medical records of neonates admitted from 2 May 2025 to 22 June 2025.

Study population

Cases

Neonates (aged ≤28 days) admitted to the hospital during the study period were diagnosed with severe neonatal jaundice. Severe NNJ was defined as requiring phototherapy or exchange transfusion based on total serum bilirubin (TSB) levels exceeding the thresholds recommended by the American Academy of Pediatrics (AAP) guidelines, adjusted for age in hours and risk factors, or according to established local hospital protocols if specific variations existed. Infants with major congenital anomalies, culture-proven sepsis at admission, or known hemolytic disease due to causes other than ABO/Rh incompatibility (hereditary spherocytosis) were excluded [14-15].

Controls

Neonates admitted to the same hospital during the same period did not develop severe jaundice. Controls were selected from infants admitted for other reasons (e.g., minor infections resolved quickly, social admissions, routine check-ups) or those with physiological jaundice whose TSB levels remained below the treatment threshold throughout their stay. Controls were frequency-matched (or state if individually matched, by age +/- sex) to cases where feasible, aiming for a ratio of 2:1 controls to cases. Exclusion criteria similar to cases regarding major anomalies and specific hemolytic conditions were applied.

Data collection

A standardized data extraction form was used to collect information from inpatient medical records. Variables collected included: A) demographics comprising of infant's sex, date of birth, and age at admission. B) Birth characteristics including the birth weight (measured within hours of birth, recorded in grams), gestational age (based on maternal dates and/or clinical assessment like Ballard score), and mode of delivery. C) Jaundice details (cases) that included recording peak total serum bilirubin (TSB) level (mg/dL or µmol/L), age at peak TSB, treatment received (phototherapy type/duration, exchange transfusion). D) Clinical factors such as Apgar scores, presence of cephalohematoma or significant bruising, and feeding type (exclusive breast, formula, mixed). E) Maternal factors such as maternal age, parity, history of maternal diabetes or hypertension, and blood group.

Variable definitions

Birth weight was categorized based on World Health Organization (WHO) criteria into three groups: low birth weight (LBW), defined as <2500 g; normal birth weight (NBW), defined as 2500-3999 g; and macrosomia, defined as ≥4000 g. Severity of jaundice was defined by the need for treatment (phototherapy or exchange transfusion) as per the criteria of the American Academy of Paediatrics [14-15]. The gestational age was categorized into preterm (<37 weeks), term (37−41 weeks and six days), and post-term (≥42 weeks). Adjust categories as relevant, late preterm (34-36 weeks and six days).

Statistical analysis

Data were analyzed using Statistical Software, SPSS version 26 (IBM Corp., Armonk, NY, USA). Descriptive statistics [frequencies, percentages, means, standard deviations (SD)] were used to characterize the case and control groups. Chi-square (χ2) test or Fisher's exact test (for cell counts <5) was used to compare categorical variables (e.g., proportion of LBW in cases vs. controls). Independent samples t-test or Mann-Whitney U test was used to compare continuous variables (mean birth weight) between groups, depending on data distribution assessed by normality tests (Shapiro-Wilk). Binary logistic regression analysis was performed to determine the association between birth weight categories (using NBW as the reference category) and severe NNJ (case vs. control status). Odds ratios (OR) and their 95% confidence intervals (CI) were calculated. A multivariate logistic regression model was used to adjust for potential confounding variables, primarily gestational age, and potentially others identified as significant in bivariate analysis (mode of delivery, maternal diabetes). A p-value <0.05 was considered statistically significant.

Ethical considerations

Ethical approval for this study was obtained from the Ethics Committee of Al-Russafa Health Directorate/Ibn Al-Balady Hospital of Children and Maternity and the relevant health directorate in Baghdad. As this was a retrospective study, patient medical records were utilized after obtaining the necessary approval. Patient confidentiality was maintained by anonymizing all data before analysis.

## Results

Table 1 shows that Neonates with severe jaundice were admitted at a younger age than controls (3.1 vs. 3.8 days; p=0.04). Gestational age was significantly lower in cases (37.2 vs. 39.0 weeks; p<0.001), with a markedly higher proportion of preterm births (28.3% vs. 5.9%; p<0.001). No significant differences were observed in sex distribution, maternal age, or cesarean delivery rates between groups. 

**Table 1:** Participant Recruitment and Baseline Characteristics p-values were calculated using an independent samples t-test for continuous variables and chi-square test (χ²) for categorical variables.

Variable	Cases (n=152)	Controls (n=305)	Test Statistic (t/χ²)	p-value
Mean Age at Admission (days)	3.1 ± 1.5	3.8 ± 2.1	t=2.03	0.04
Male Sex (%)	94 (61.8%)	177 (58.0%)	χ²=0.68	0.41
Mean Gestational Age (weeks)	37.2 ± 1.9	39.0 ± 1.4	t=10.1	<0.001
Preterm Births (<37 weeks)	43 (28.3%)	18 (5.9%)	χ²=45.6	<0.001
Mean Maternal Age (years)	28.5 ± 5.5	29.1 ± 6.0	t=0.94	0.35
Cesarean Delivery (%)	64 (42.1%)	112 (36.7%)	χ²=1.41	0.23

As shown in Tables 2, 3 (panels A & B), neonates with severe jaundice had significantly higher peak total serum bilirubin compared with controls (23.5 vs. 13.8 mg/dl; p<0.001), with nearly all exceeding phototherapy thresholds (96.1% vs. 17.0%) and about one-fifth requiring exchange transfusion (21.1% vs. 0.7%; p<0.001). Cases reached peak bilirubin earlier and had higher direct bilirubin levels than controls (p<0.001 for both). Among cases, all received phototherapy, with an average duration of 72 hours; 19.7% underwent exchange transfusion, 7.9% received intravenous immunoglobulin (IVIG), and 31.6% required neonatal intensive care unit (NICU) admission, with a mean hospital stay of 6.2 days.

**Table 2:** Bilirubin Levels and Treatment, Panel A - Bilirubin Metrics (Cases vs Controls) TSB: total serum bilirubin ; SD: standard deviation; IQR: inter quaterile range.

Variable	Cases (n=152)	Controls (n=305)	Test (t/χ²)	p-value
Peak TSB, mg/dL (mean ± SD)	23.5 ± 4.2	13.8 ± 3.1	t=27.9	<0.001
Peak TSB, median (IQR)	23.0 (20.0–26.0)	14.0 (12.0–16.0)	—	—
Age at peak TSB, hours (mean ± SD)	80 ± 18	90 ± 20	t=5.10	<0.001
Direct bilirubin, mg/dL (mean ± SD)	1.8 ± 0.7	1.2 ± 0.5	t=9.32	<0.001
Exceeded phototherapy threshold n (%)	146 (96.1%)	52 (17.0%)	χ²=301.4	<0.001
Exceeded exchange transfusion threshold n (%)	32 (21.1%)	2 (0.7%)	χ²=61.8	<0.001

**Table 3:** Panel B - Treatment Among Cases Only p-values were calculated using an independent samples t-test for continuous variables and chi-square test (χ²) for categorical variables. IVIG: intravenous immunoglobulin; NICU: neonatal intensive care unit.

Treatment / Outcome	Cases (n=152)
Any phototherapy, n (%)	152 (100%)
Duration of phototherapy (hours, mean ± SD)	72 ± 24
Exchange transfusion, n (%)	30 (19.7%)
IVIG, n (%)	12 (7.9%)
NICU admission, n (%)	48 (31.6%)
Length of stay, days (mean ± SD)	6.2 ± 2.1

Table 4 presents the analysis of birth weight distribution, revealing that low birth weight (<2500 g) was significantly more common among cases compared with controls (25.7% vs. 9.2%; χ²=26.8, p<0.001). Normal birth weight (2500-3999 g) was the predominant category in both groups, but was more frequent among controls (85.6% vs. 69.1%). Macrosomia (≥4000 g) was almost identical between groups (5.3% vs. 5.2%; p=0.96), showing no association with severe jaundice. Overall, the distribution of birth weight categories differed significantly between cases and controls (χ²=27.1, p<0.001).

**Table 4:** Birth Weight Category Distribution p-values were calculated using Pearson’s chi-square test.

Birth Weight Category	Cases (n=152)	Controls (n=305)	Test Statistic (χ²)	p-value
Low Birth Weight (<2500 g)	39 (25.7%)	28 (9.2%)	χ²=26.8	<0.001
Normal Birth Weight (2500–3999 g)	105 (69.1%)	261 (85.6%)	—	—
Macrosomia (≥4000 g)	8 (5.3%)	16 (5.2%)	χ²=0.002	0.96
Overall (df=2)	—	—	χ²=27.1	<0.001

Table 5 shows that logistic regression analysis demonstrated that low birth weight (<2500 g) was strongly associated with severe neonatal jaundice. In the unadjusted model, LBW neonates had 3.45 times higher odds of developing severe jaundice compared to those with normal birth weight (95% CI: 2.08-5.71; p<0.001). After adjusting for gestational age, this association remained significant [adjusted odds ratio (AOR)=2.78; 95% CI: 1.59-4.86; p<0.001]. In contrast, macrosomia (≥4000 g) showed no significant association in either the crude or adjusted models. Gestational age was identified as a protective factor, with each additional week reducing the odds of severe jaundice by approximately 35% (AOR=0.65; 95% CI: 0.57-0.74; p<0.001).

**Table 5:** Logistic Regression Analysis for Severe Neonatal Jaundice Binary logistic regression analysis was used. The adjusted model includes gestational age as a covariate. OR: odds ratio; CI: confidence interval.

Predictor	Crude OR (95% CI)	p-value	Adjusted OR (95% CI)†	p-value
Low Birth Weight (<2500 g)	3.45 (2.08 – 5.71)	<0.001	2.78 (1.59 – 4.86)	<0.001
Macrosomia (≥4000 g)	1.01 (0.43 – 2.36)	0.99	1.15 (0.48 – 2.77)	0.75
Gestational Age (per week)	—	—	0.65 (0.57 – 0.74)	<0.001

## Discussion

This case-control study conducted at a major pediatric referral hospital in Baghdad found a significant association between low birth weight (<2500 g) and the development of severe neonatal jaundice requiring treatment. Infants born with LBW had nearly three times the odds of developing severe NNJ compared to infants with normal birth weight, even after adjusting for the strong confounding effect of gestational age.

This finding aligns with established pathophysiology and previous research from various global settings [16-17]. LBW infants, particularly those who are also preterm or small for gestational age, often exhibit hepatic immaturity, leading to reduced activity of the UDP-glucuronosyltransferase (UGT1A1) enzyme crucial for bilirubin conjugation [16]. They may also experience delayed establishment of effective feeding, leading to increased enterohepatic circulation of bilirubin, and potentially have higher rates of bilirubin production relative to their body mass [18-19]. Our results, demonstrating the persistence of this association after controlling for gestational age, suggest that low birth weight itself, independent of prematurity alone, contributes to the risk within this population.

The lack of a significant association between macrosomia and severe NNJ in our cohort contrasts with some reports [20] but is consistent with others [21]. Potential reasons for inconsistency in the literature include varying definitions of macrosomia, differences in the prevalence of associated factors like maternal diabetes or birth trauma, cephalohematoma, bruising between study populations, and statistical power limitations. In our setting, it appears macrosomia is not a primary independent risk factor for severe jaundice requiring admission and intervention, although it might contribute in specific individual cases secondary to birth trauma.

Our findings of LBW and preterm associated with severe hyperbilirubinemia align with a meta-analysis done on low- and middle-income countries, which found that low birth weight and preterm are both associated with severe neonatal jaundice needing active management [11].

This case-control study leveraged routine clinical data and standardized definitions to quantify the association between birth weight and severe NNJ. Strengths include a relatively large sample, clear inclusion criteria, and adjusted analyses that account for gestational age. However, its retrospective design may introduce selection and information bias; unmeasured confounders (hemolysis etiologies, feeding dynamics) could persist; and variable missingness necessitated complete-case sensitivity analyses. These factors are unlikely to overturn the primary finding that LBW and lower gestational age are strong risk markers for severe NNJ, but they may attenuate secondary associations.

To prevent severe jaundice in neonates and its complications, we recommend identifying babies with high-risk factors for severe neonatal jaundice before discharge, including LBW, preterm, family history of neonatal jaundice needing active management, visible jaundice in the first 24 hours, blood group incompatibility, sepsis, and other risk factors. We also recommend that the baby be checked for jaundice by the parents, carer, and healthcare professionals in the first few days of life. However, sometimes even neonatologists can miss neonatal jaundice at its beginning; therefore, we recommend using transcutaneous bilirubin (TCB) measurement for all babies with high-risk factors and then proceeding to measure total serum bilirubin (TSB) when the result of the TCB test indicates that (at the threshold) [13, 22]. It is proven that using a screening programme for neonatal hyperbilirubinemia is associated with a decreased incidence of severe hyperbilirubinemia; however, it is also associated with more use of phototherapy [14].

We did not find an online Iraqi screening and management guideline for neonatal jaundice, and we do not have a specific screening programme; and usually depend on the National Institute for Health and Care Excellence (NICE) guidance and other international guidance, which leads to differences in the management approach. Therefore, we recommend the establishment of national Iraqi guidance for this context to improve the quality of care. 

## Conclusions

Our findings shed light on several important factors that may increase a newborn’s risk of developing severe neonatal jaundice. Babies who were born preterm or with low birth weight were especially vulnerable, and these characteristics were consistently more common among affected infants. Even though phototherapy was widely used and effective in most cases, some newborns still needed exchange transfusion, which highlights how serious this condition can become without timely care. We also found that babies delivered by cesarean section were more likely to develop severe jaundice, though the reason for this needs further exploration. Interestingly, as babies spent more time in the womb, their risk of severe jaundice decreased, reinforcing the protective effect of gestational maturity. Taken together, these results emphasize the importance of early monitoring and intervention, especially for preterm and low birth weight infants-to help prevent severe outcomes and ensure safer neonatal care.
